# Development of Neurodegenerative Disease Diagnosis and Monitoring from Traditional to Digital Biomarkers

**DOI:** 10.3390/bios15020102

**Published:** 2025-02-11

**Authors:** Jaeyoon Song, Eunseo Cho, Huiseop Lee, Suyoung Lee, Sehyeon Kim, Jinsik Kim

**Affiliations:** Department of Biomedical Engineering, College of Life Science and Biotechnology, Dongguk University, Seoul 04620, Republic of Korea; thdwodbs95@dongguk.edu (J.S.); 2021111733@dgu.ac.kr (E.C.); 2018111717@dgu.ac.kr (H.L.); suyoung7541@dongguk.edu (S.L.); kshsarah1@gmail.com (S.K.)

**Keywords:** digital biomarker, neurodegenerative disease, traditional biomarker, monitoring, point of care

## Abstract

Monitoring and assessing the progression of symptoms in neurodegenerative diseases, including Alzheimer’s and Parkinson’s disease, are critical for improving patient outcomes. Traditional biomarkers, such as cerebrospinal fluid analysis and brain imaging, are widely used to investigate the underlying mechanisms of disease and enable early diagnosis. In contrast, digital biomarkers derived from phenotypic changes—such as EEG, eye movement, gait, and speech analysis—offer a noninvasive and accessible alternative. Leveraging portable and widely available devices, such as smartphones and wearable sensors, digital biomarkers are emerging as a promising tool for ND diagnosis and monitoring. This review highlights the comprehensive developments in digital biomarkers, emphasizing their unique advantages and integration potential alongside traditional biomarkers.

## 1. Introduction

Neurodegenerative diseases (NDs) are brain-related disorders characterized by the progressive loss of neurons along with structural and functional damage to the brain caused by aging. Including Alzheimer’s disease (AD), Parkinson’s disease (PD), amyotrophic lateral sclerosis (ALS), frontotemporal lobar degeneration (FTLD), Huntington’s disease (HD), prion disease, and dementia, NDs have become more common due to population aging, posing a significant public health challenge with increasing social and medical burdens. AD is a most common ND in which changes in biological substances such as Aβ and tau proteins lead to cognitive decline, while PD leads to motor and non-motor symptoms due to the aggregation of α-synuclein [1,2]. Globally, over 50 million individuals are impacted by NDs, and projections indicate that this figure is anticipated to nearly triple, reaching 152 million by the year 2050. The diseases associated with cognitive decline, severe motor impairments, and social dysfunction lead to difficulties in daily life [3,4].

NDs have clinically been diagnosed using questionnaires such as the Mini-Mental State Examination (MMSE) and Parkinsons’ Disease Questionnaire-39 (PDQ-39), as well as assessments of behavioral abilities [5,6]. However, traditional diagnostic methods have limitations, including difficulty in accurately diagnosing diseases, non-specificity, and subjectivity [7]. Advancements in precise diagnostics for NDs have been made to overcome these limitations, incorporating methods such as cerebrospinal fluid (CSF) biomarkers and brain imaging.

Traditional biomarkers and brain imaging serve as indicators to evaluate normal biological processes, pathological processes, and drug treatment interventions in NDs [8]. Body fluid-based traditional biomarkers could detect differences signifying diseases by analyzing changes in microRNA, peptides, molecules, or protein levels in blood and CSF. Additionally, brain imaging such as Fluorodeoxyglucose (FDG)-PET, amyloid-PET, and fMRI can monitor disease progression and enable early diagnosis of AD and PD [9,10].

However, traditional biomarkers may not be specific to certain diseases and could have limitations in accurate diagnosis. Since traditional biomarkers relate to various physiological mechanisms, analyzing one or a few biomarkers may cause inaccurate diagnoses, such as false positives or negatives. Therefore, traditional biomarkers cannot conclusively diagnose diseases using only one or a few biomarkers [11,12]. In addition, methods for diagnosing NDs with traditional biomarkers are limited as they require specific facilities that are only available in hospitals or laboratories, and they require high costs, lengthy analysis time, and expensive equipment. Therefore, traditional biomarkers have limitations in continuous monitoring and early diagnosis of NDs.

Traditional biomarkers represent biological substances such as amyloid beta, tau, dopamine, and brain imaging accordingly, whereas digital biomarkers refer to biomarkers that can analyze changes in behavior and symptoms that may come from patients with NDs using digital healthcare technologies [13]. Digital biomarkers have recently been highlighted as biomarker candidates, consisting of digital data used to collect and analyze physiological signals using sensors or digital devices. Physiological signals vary in daily life, making the analysis of digital biomarkers crucial for facilitating real-time monitoring and diagnosis [14]. These digital biomarkers can be measured in various formats, such as electrograms, eye movements, and gait, and are used to diagnose NDs like AD and PD [15]. Digital biomarkers have the advantage of enabling quantitative and noninvasive measurements of NDs through simple devices that can be portable, wearable, and contactless, unlike traditional biomarkers. In addition, they provide a means to monitor patients’ physical abilities and diseases, enabling remote and continuous data collection for early diagnosis of diseases and disease management [16,17,18]. Based on digital biomarkers, personalized approaches to disease monitoring and management can be significantly improved.

This review focuses on recent studies using traditional and digital biomarkers for diagnosing ND. The definition of ND is first examined to utilize these biomarkers, focusing on AD and PD. The review discusses traditional biomarkers used for diagnosing AD and PD, such as CSF biomarkers and brain imaging, and outlines digital biomarker types recently highlighted in research. In this study, the applicability and future development of both traditional and digital biomarkers for the diagnosis and prediction of ND will be discussed.

## 2. Overview of Neurodegenerative Disease: Alzheimer’s Disease (AD) and Parkinson’s Disease (PD)

NDs involve the gradual deterioration of nerve cells in the central nervous system, resulting in symptoms such as dementia, cognitive impairment, and reduced motor function [8,19,20,21]. This impacts core functions like memory, behavior, language, and emotional regulation, often resulting in chronic cognitive decline and significantly affecting daily life [22,23]. There are various types of ND, including AD and PD, which are the most common NDs. Importantly, this neurodegeneration typically manifests progressively with age, leading to an increased incidence of conditions like AD and PD as individuals get older.

AD is one of the most common NDs, caused by a cascade of pathophysiological changes, such as aggregation of Aβ peptides into Aβ plaques, hyperphosphorylation and aggregation of tau proteins, and formation of neurofibrillary tangles. The pathogenic process of AD decreases synaptic function, resulting in symptoms such as neurodegeneration and cognitive decline, depressed feelings, language and behavioral impairments, and disorientation [24]. Since the major neuropsychiatric symptoms include depression, aggression, and loss of appetite, depending on the progression of the disease, and it does not have efficient treatment methods, the early diagnosis and monitoring of progression are sufficient [25,26].

These symptoms of AD have been traditionally diagnosed using questionnaires and behavioral assessments such as the MMSE, Geriatric Depression Scale (GDS), and Neuropsychiatric Inventory Questionnaire (NPI-Q) [5]. However, diagnosing AD with these questionnaires has a limited ability to distinguish healthy groups from patients with mild AD and to identify specific functional impairments. Additionally, diagnosing AD based on behavioral assessments is non-specific and subjective, making them insufficient for accurate diagnosis [7,27].

PD is an ND in which α-synuclein aggregation progresses gradually; nerve cell damage occurs, and dopamine imbalance results in dysfunction [28,29,30]. Patients with PD have both motor and non-motor symptoms, which appear from the beginning of the disease, affecting patients’ quality of life [31]. Major motor symptoms such as tremors, unstable posture, muscle weakness, and oral movement disorders appear due to the onset of Parkinson’s disease. Additionally, non-motor symptoms such as drowsiness, constipation, loss of smell and taste, and sleep abnormalities appear [32].

PD has traditionally been diagnosed based on motor and non-motor symptoms by using methods such as the Unified Parkinson’s Disease Rating Scale (UPDRS), Hoehn and Yahr scale, and PDQ-39 [6]. However, similar to the traditional diagnosis of AD, the diagnostic process for PD using these questionnaires also faces clear limitations [33].

**Figure 1:**
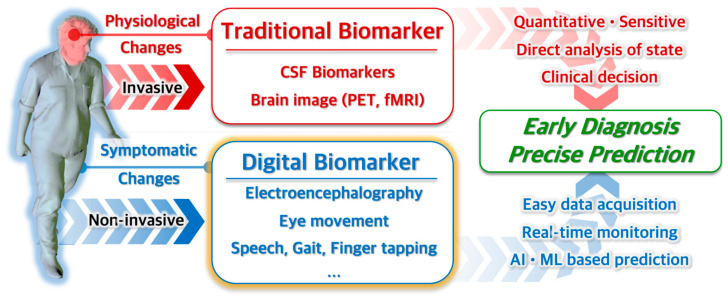
Role of traditional and digital biomarkers in early diagnosis of ND.

To overcome the limitations of existing diagnostic methods, efforts have been made to develop new biomarkers (Figure 1). CSF biomarkers and brain imaging analysis are being explored to create objective, quantifiable, and highly sensitive diagnostic methods. These are currently considered the most reliable and are used alongside traditional clinical questionnaires for diagnosing ND. As the number of patients with NDs has increased and the diseases progress chronically, there has been a significant increase in research focused on wearable devices for monitoring ND in daily life or digital biomarkers that can diagnose based on changes in everyday activities.

## 3. Traditional Biomarkers in ND Diagnosis

Studying traditional biomarkers is a valuable tool for scientific research, including disease diagnosis, mechanism analysis, treatment development, and evaluation. Traditional biomarkers have several advantages for diagnosing ND. One of the most significant benefits is that they allow for precise and quantitative biological analysis of the disease. Therefore, they are valuable for early disease detection, diagnosis, disease monitoring, and tracking the progression of disease since they can be measured quantitatively [34,35]. By using traditional biomarkers such as genotype, CSF biomarkers, and brain imaging, changes in RNA, proteins, or molecules within the body can be measured for diagnosing ND (Table 1).

CSF biomarkers such as amyloid beta, tau, BACE1 and α-synuclein are used for diagnosing ND. The levels and ratio of amyloid beta 42 and amyloid beta 40 in CSF are used to diagnose AD and PD [29,38,42,43,46,60,61,62]. Also, changes in the levels of p-tau, t-tau, BACE1, and α-synuclein are utilized for diagnosis [39,40,41]. These CSF biomarker-based diagnosis methods have been widely utilized because they directly analyze biomarker levels in the CSF, providing high accuracy. Additionally, CSF biomarkers provide direct information about diseases, enabling early diagnosis and monitoring for the progression of diseases. Diagnosis and prediction using genotyping, not only CSF biomarkers, are being conducted. Genotyping can confirm familial AD primarily by analyzing apolipoprotein E (APOE) mutations. This can help assess the likelihood of MCI patients progressing to AD based on their genotype and family history [29,36,37].

Brain imaging can also be used to diagnose ND. Imaging methods such as resting-state fMRI (rs-fMRI) and FDG-PET allow for the analysis of brain networks and the evaluation of metabolic activity, enabling the assessment of functional and structural abnormalities in the brain [55,56,57,58,59,75]. Amyloid beta, neurofilament light chain, and dopamine are utilized in CSF biomarker analysis and brain imaging, facilitating disease-state monitoring. Furthermore, the combined use of CSF biomarkers and brain imaging can enhance the accuracy of ND diagnosis.

Traditional biomarkers, such as CSF biomarkers and brain imaging, can be used to diagnose ND early. Diagnosing ND using CSF biomarkers and brain imaging can analyze a direct correlation between biomarker level and disease progression to predict the disease early. If an ND is diagnosed using CSF analysis and brain imaging, the progress of the disease can be monitored [76,77,78,79,80]. However, when using CSF biomarkers, there are limitations, such as being invasive and having low specificity when relying on a single biomarker [12]. Additionally, CSF biomarkers and brain imaging heavily depend on expensive facilities, such as ELISA, MRI, and PET equipment [81,82]. Therefore, the need to develop affordable biomarkers that allow cost-effective, noninvasive, continuous daily-life monitoring is emerging along with traditional biomarkers.

## 4. Digital Biomarkers for Advanced ND Diagnosis

Digital biomarkers offer various advantages compared to traditional biomarkers, such as affordability, noninvasive analysis, potential for continuous and remote monitoring, and easy data acquisition in everyday life. These advantages enable early diagnosis and prognosis analysis [18,81,83,84,85]. As described in Table 2, potential digital biomarkers are currently being widely researched and measured with wearable, handy, simple devices [18,86].

This simplified and streamlined measurement approach highlights the unique advantages of digital biomarkers over traditional biomarker analysis. As previously discussed, traditional biomarkers require bulky, expensive equipment and testing methods. For instance, APOE genotype analysis involves PCR testing with blood samples, CSF biomarkers necessitate enzyme-linked immunosorbent assays (ELISAs) or mass spectrometry (MS), and brain imaging relies on MRI and PET scans, which are restricted to specialized facilities and incur high costs. In contrast, digital biomarkers present an alternative that circumvents these limitations by using wearable electrodes, smartphones, cameras, smartwatches, and other portable devices. These tools facilitate seamless data collection in daily life, making digital biomarkers a more accessible and cost-effective means for monitoring health conditions.

Methods such as electroencephalogram (EEG), the most researched analysis method in ND diagnosis, along with sleep behavior, driving behavior, eye movement, gait, finger tapping, and speech analysis, could also be used for ND diagnosis. These analytical methods relate to cognitive and motor functions, facilitating the diagnosis of ND. Additionally, these methods are suitable for real-time monitoring thanks to portable, compact measurement devices. In particular, gait and speech analysis could be conducted with a smartphone or other small devices in daily use; their real-time monitoring capabilities are significant. Contrastingly, task-dependent methods such as sleep and driving behavior analysis are still in development due to their limitation of measurement in daily life.

Achieving the developed diagnosis of ND based on these digital biomarkers requires feature extractions from captured data to develop them as digital biomarkers, which are sufficient. The extracted digital biomarkers could diagnose ND through computational analysis, including machine learning (ML)- or deep learning (DL)-assisted algorithms.

Digital biomarkers provide a convenient means of continuously monitoring disease progression and severity by monitoring patients’ activities. Based on these biomarkers, personalized approaches to disease management and patient outcomes can be significantly enhanced. Therefore, digital biomarkers can potentially develop highly accurate, personalized, real-time-monitoring-capable prediction models for ND.

In this section, we reviewed recent studies about digital biomarkers based on the analysis methods in Table 2, and found their capabilities in early diagnosis, real-time monitoring, and personalized diagnosis with ML- and DL-assisted modeling.

### 4.1. Electroencephalogram

One of the most widely utilized digital biomarkers for ND is EEG. It garners attention due to its widespread availability, noninvasiveness, and ability to monitor and analyze brainwave signals in real time across various environments compared to traditional biomarkers. Nowadays, EEG can be measured quantitatively, which is known as quantitative EEG. The results of these measurements can be used to extract various EEG features and utilize them as digital biomarkers. The features, including spectra, relative power, complexity, connectivity, spindle, and so on, can be analyzed to differentiate ND patients from HCs and disease subtypes using various analysis methods and machine learning-based analysis [87,90,91,92,93].

Brainwave signals can be divided into several frequency bands through signal processing, such as Fourier transform, wavelet transform, and autoregressive methods, reflecting various brain functions [94]. Consequently, spectral analysis of EEG can be utilized to predict the brain’s physiological state and assist in diagnosing ND. Aparisi et al. measured the resting EEG of PD patients and confirmed the relative power, and its ratio of EEG can be utilized in the early detection of PD [95]. The researchers measured the patient’s EEG while the eyes were closed, opened, and in a reactive state. The results showed that the quantitative resting EEG can discriminate the PD from the HC, and the efficient EEG biomarkers differed due to the measurement conditions.

Additionally, the feature extraction methods are different from the results in EEG analysis. Chang et al. applied Holo-Hilbert Spectral Analysis (HHSA) methods to reveal nonlinear features of resting EEG [96]. They measured resting EEG in 99 PD and 59 HC patients. The analyzed EEG band relative power through HHSA feature extraction resulted in high accuracy in the identification of late-stage PD and early-stage PD with 90% accuracy. Therefore, spectral analysis of EEG can be utilized in the prediction and diagnosis of PD and also in monitoring the prognosis.

**Figure 2:**
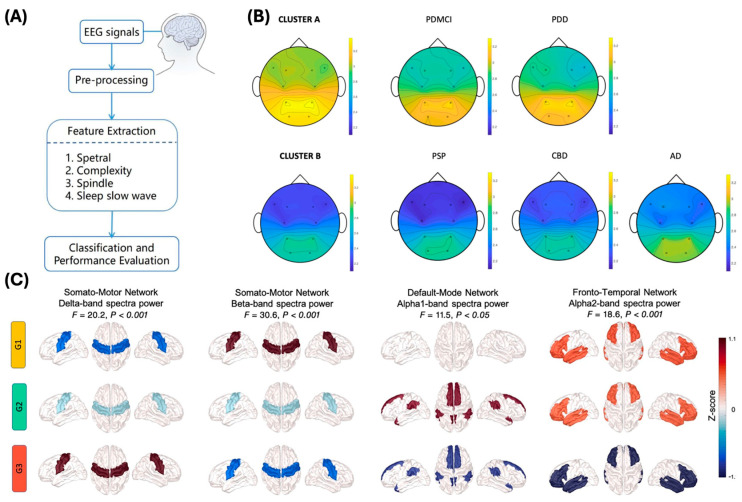
EEG analysis for ND diagnosis. (**A**) Example of EEG feature analysis process in diagnosis [87]. (**B**) EEG complexity-based ND diagnosis. The topographical distribution of the power-law exponent β shows the difference between ND types. Cluster A: α-synucleinopathies; Cluster B: tauopathies; PDMCI: PD with MCI; PDD: PD with dementia; PSP: progressive supranuclear palsy; CBD: corticobasal degeneration [88]. (**C**) Spectra power analysis of EEG band for the functional region of the brain. The relative power and the functional connectivity can differentiate disease subtypes of PD. G1: moderate, with rapid motor progression; G2: moderate; G3: mild to severe [89].

Moreover, the signal complexity and functional connectivity of EEG can be utilized in diagnosing ND. Since the EEG signals represent the functionalities of each brain region and physiological state, and the neural network works with complex connections to perform such functions, the complexity and connectivity analysis are highlighted as digital biomarkers of ND [97,98]. Because of the complexity of those signals, various approaches to analyzing them were tried, including machine learning-based analysis. Mostile et al. recently showed EEG-based differentiation of ND with complexity analysis [88]. They measured EEG from 230 patients with a diagnosis of tauopathy (AD, PSP, CBD) or α-synucleinopathies (PDNC, PDMCI, PDD). The measured signal was analyzed using spectral analysis methods and computed in β values to represent complex neural activity. From the calculated β values, the results showed that the power-law exponent β could be utilized to differentiate ND types, even in the subtypes of each pathology (Figure 2B). Yassine et al. reported a 5-year follow-up study to confirm that resting-state EEG can be utilized in PD diagnosis and to discriminate PD subtypes [89]. They measured high-density EEG (HD-EEG) in 44 PD patients for 5 years and analyzed EEG signals with relative power and the functional connectivity of each frequency band. In Figure 2C, the researchers showed the comparison of relative power and functional connectivity can subgroup PD into a moderate group with rapid motor progression (G1), a moderate group (G2), and a mild-to-severe group (G3). The results show that the EEG spectral and connectivity features can be used in the early diagnosis of PD.

The feature extraction methods were also important due to the complexity of EEG signals. Vicchietti et al. confirmed promising results for detecting AD through EEG analysis [99]. They acquired EEG signals from 160 AD patients and 24 HCs and analyzed them using six time-series analysis methods, including wavelet coherence, fractal dimension, quadratic entropy, wavelet energy, quantile graphs, and visibility graphs. They showed that when utilizing delta waves, the results achieved very low p-values and high AUC values close to 1.0, achieving the highest accuracy of 98% in distinguishing between AD and HC.

Through the development of EEG-based ND diagnosis, machine learning and computational prediction can be conducted to enhance accuracy and enable point-of-care systems. Amato et al. analyzed functional connectivity-based EEG to diagnose MCI and proposed a personalized prediction model for the early diagnosis of AD [100]. They collected EEG records from HC, SCD, and MCI groups and examined the EEG biomarkers. The EEG analysis can distinguish MCI from HCs with 78% accuracy by analyzing functional connectivity and power spectral density. The analysis predicted the SCD subgroup, achieving 48% accuracy in classifying MCI and SCD versus HC groups, which yielded positive and negative results in CSF biomarker analysis. They also developed a personalized prediction model using a computational AD model, demonstrating 95% accuracy in MCI discrimination and 87% in SCD discrimination HC. Through these development approaches in EEG analysis for personalized diagnosis and monitoring, EEG is one of the most researched digital biomarkers alongside other digital biomarkers in ND diagnosis.

### 4.2. Eye Movement

**Figure 3:**
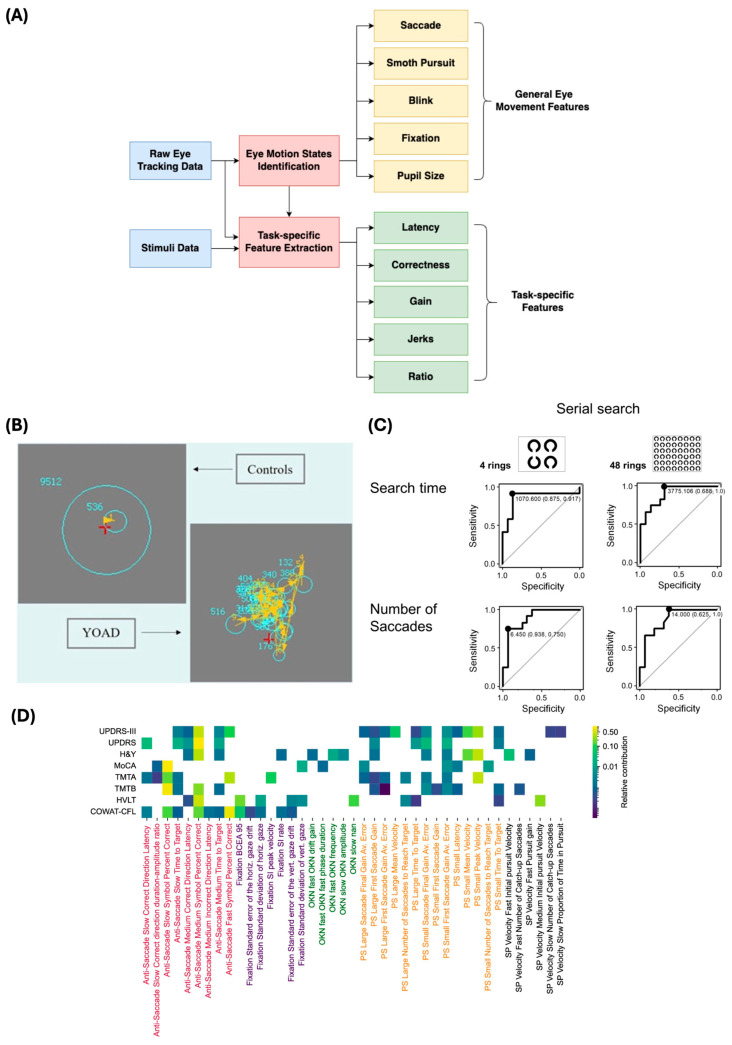
Eye movement analysis for ND diagnosis. (**A**) Examples of eye movement digital biomarkers. Reprinted from ‘Automating the analysis of eye movement for different neurodegenerative disorders’, Vol 170, Li et al., 107951, Copyright 2024, with permission from Elsevier [105]. (**B**) Eye fixation stability results of HCs and young-onset AD (YOAD) [106]. (**C**) Diagnosis performance of visual search task results in AD patients [107]. (**D**) Correlation heatmap between eye movement digital biomarkers and a clinical score of PD [108].

Eye movement analysis is also a well-known digital biomarker of ND. For decades, research has validated the effectiveness of eye imaging and tracking in diagnosing ND [109,110,111,112,113,114,115]. Pupillometry, which measures pupil size and reactivity, is commonly used in clinical decisions of ND diagnosis, and it has led to emerging research into eye movement analysis in ND [116,117]. Eye movement biomarkers can be categorized into general eye movement features and task-specific features [105], and those features have been analyzed under various conditions, such as tracking tasks, finding tasks, or light reflex changes (Figure 3A).

For example, Pavisic et al. measured fixation stability pro-saccade and smooth pursuit metrics from 36 YOAD patients and 21 HCs [106]. Including eye fixation stability in Figure 3B, there was a significant difference in eye movement digital biomarkers observed and the ML-based prediction model could identify YOAD from HCs with an accuracy of 95%. Tokushige et al. investigated eye movements to detect cognitive decline in the early stage of AD [107]. The researchers measured the eye movement of 16 AD patients and 16 HCs with visual memory and search tasks. After analyzing eye tracking under specific tasks, they could identify AD from HCs with high sensitivity and specificity. Eye movement features are also widely utilized in PD diagnosis since they relate to cognitive and motor functions. Koch et al. recently reported eye movement capture via an electronic tablet and validated that it can discriminate PD patients from HCs as a fast, easy, and cost-effective method [108]. The researchers measured eye movement with an iPad Pro tablet, gave visual stimuli on screen, and captured them simultaneously. In Figure 3D, the researchers analyzed correlations between analyzed features and clinical score of PD. With those features, they could be classified as mild PD, moderate PD, and severe PD with an AUC of 0.94. Through the development of eye movement analysis, eye tracking can be a powerful digital biomarker of ND and increase the potential of real-time monitoring in daily life to predict ND early.

### 4.3. Gait

**Figure 4:**
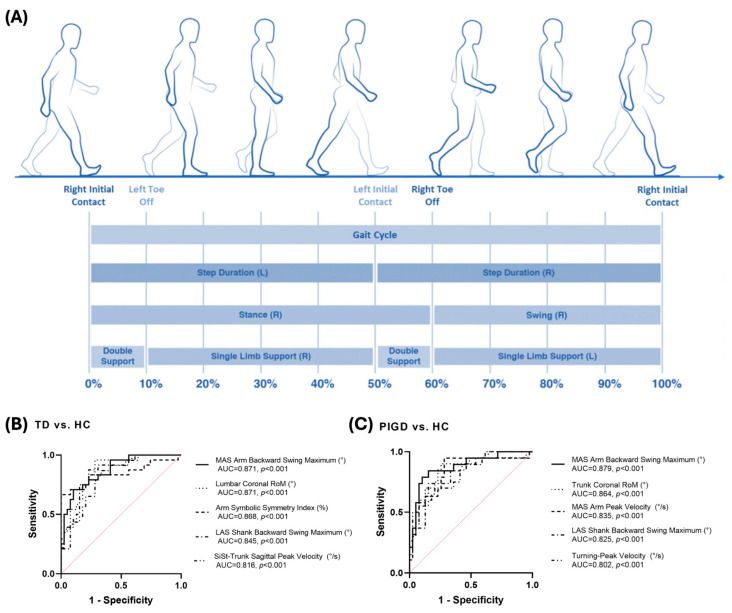
Gait analysis for ND diagnosis. (**A**) Gait cycle and detailed features applicable as digital biomarkers [118]. (**B**,**C**) Gait analysis based on PD diagnosis results. Gait analysis can identify PD from HCs and the subtypes of PD. TD: tremor-dominant; PIGD: postural instability gait difficulty [119].

Since gait is related to motor function, gait-based analysis is a promising method for diagnosing ND, particularly PD. As motor and cognitive functions gradually decline with the progression of ND, gait dysfunctions emerge, which can be utilized for diagnosis and prediction [120,121,122,123,124,125,126]. Gait biomarkers can be extracted from the gait cycle and the movement of the arm in between gait cycles. Then, various gait biomarkers can be analyzed to conduct ND diagnosis (Figure 4A) [118]. Since the gait can be measured with wearable sensors, gait analysis has the potential for real-time monitoring in daily life. For example, Ellis et al. measured stride time and stride length with a smartphone to analyze the gait variability of PD patients [127]. They used an accelerometer and gyroscope in a smartphone to measure gait variability and analyze the stride time and length of 12 PD patients and 12 HCs. The results showed a clear difference in gait variability to the proposed potentials of gait analysis-based PD diagnosis in daily life. Ghosal et al. applied gait monitoring in daily life to validate AD diagnosis capability [17]. They attached accelerometers to AD patients and HCs for continuous gait-related monitoring over 7 days. They classified the acquired monitoring data into five characteristics of gait: amplitude, speed, rhythm, symmetry, and variability. As a result, they confirmed the correlation between gait metrics and cognitive function and confirmed the distinguishability between AD and HCs. However, their correlation indicated the limitations of using solely digital biomarkers for diagnosis, with a maximum R square of 0.441.

These results showed that although the gait features were related to ND, gait-based ND diagnosis still needs to be studied. To overcome the limitations in gait analysis, Zhang et al. proposed quantitative gait analysis with wearable sensor-based gait monitoring to develop object gait biomarkers to apply to early diagnosis and subtype differentiation and severity monitoring [119]. With wearable sensors, they analyzed gait features from 24 TD patients, 20 PIGD patients, and 39 HCs. They defined gait biomarkers of PD in four categories: upper limb, lower limb, trunk and lumbar, and postural transition. With the categorized gait biomarkers, the researchers achieved a high AUC value of 0.7~0.9 in the discrimination of PD subtypes from HCs. They showed the potential of gait-based PD prediction and monitoring in daily life.

### 4.4. Finger Tapping

Finger tapping has recently been highlighted as an affordable digital biomarker in ND [129,130,131,132]. Like gait variability and balance, which are affected by motor dysfunction and cognitive impairment in neurodegeneration, finger-tapping analysis is based on the same principle as gait analysis [133]. Therefore, fine movement analysis in finger tapping could be a potential biomarker of ND.

However, quantitative and standardized finger-tapping analysis is still needed. With this need, Roalf et al. compared differences in finger tapping, inter-tap interval, and the variability in AD, PD, MCI, and healthy older adults (HOAs) [134]. They compared finger-tapping biomarkers in 131 AD, 63 PD, and 46 MCI patients, as well as 62 HOAs. All patient groups showed more impairment compared to HOAs. The AD and MCI groups showed slower tapping and longer intervals, and PD groups showed faster tapping and shorter intervals than HOAs. As a result, the finger-tapping analysis could discriminate PD patients from HOAs with an AUC of 0.89; it could also discriminate PD from AD with an AUC of 0.78.

Because finger-tapping capture does not require large facilities or a clinical measurement environment, it can be analyzed with hands-on devices such as smartphones, tablets, and cameras. In this background, Broeder et al. developed a smartphone-based tapping task and utilized it for PD medication monitoring [135,136]. A total of 32 PD patients performed the task before taking medication, followed by two tests after 1 and 3 h, repeated over the course of a week. After analyzing the tapping features, frequency, and interval, the results show that the medication dose shows a correlation with tapping feature changes, which allows the utilization of finger tapping as a monitoring biomarker of PD severity and medication responses. Moreover, Yang et al. recently developed a deep learning-assisted video-based online system named FastEval Parkinsonism to monitor the all-day variability of PD patients [137]. The researchers captured 840 finger-tapping videos from 103 PD patients, 24 atypical parkinsonism (APD) patients, 12 elderly people with mild parkinsonism signs (MPSs), and 47 HCs. The deep learning model achieved acceptable accuracies (AACs) of over 80% and demonstrated the usability of multi-angle videos of 300 PD patients from an external database. This research shows the potential of daily monitoring of PD with hands-on devices, making the monitoring and prediction of ND easier.

### 4.5. Speech

**Figure 5:**
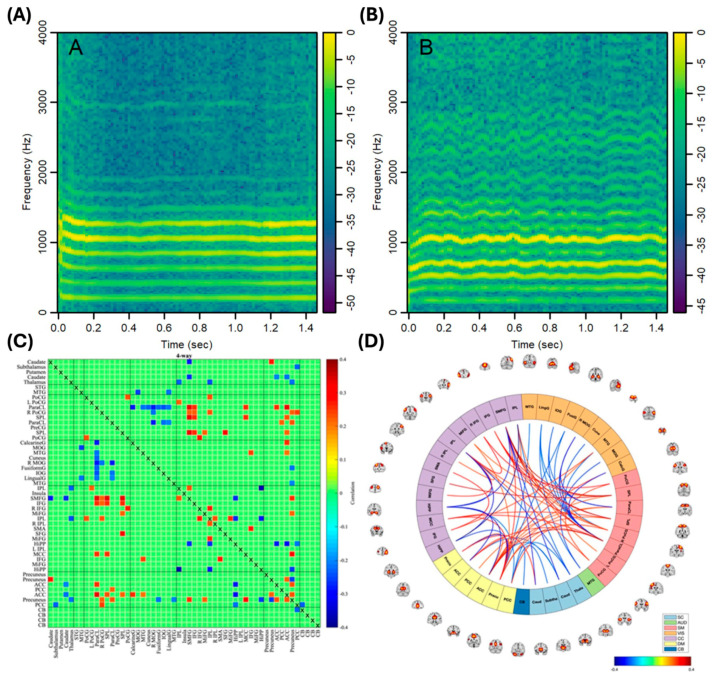
Speech analysis for ND diagnosis. (**A**,**B**) Speech variability of HCs in A and PD in B [138]. (**C**,**D**) Correlation of ML-based analyzed speech biomarkers and brain connectivity derived with CSF biomarkers and rs-fMRI measurement [16].

Along with the various digital biomarkers that provide information on cognitive function and motor function, speech analysis is also a promising biomarker of ND [139,140,141,142,143,144,145]. The speech biomarkers include acoustic features and linguistic features, which were related to patients’ cognitive and motor functions. Speech biomarkers also have advantages and are easy to acquire because they can be captured with handy devices such as smartphones. Iyer et al. utilized smartphones to collect speech-based digital biomarkers to diagnose PD patients [138]. Using signal processing techniques, they analyzed voice features and estimated acoustic signal characteristics for sustained vowel /a/ pronunciation, as shown in Figure 5A,B. They then conducted a spectrum-based analysis to classify PD patients and HCs, achieving an average AUC of 0.96 for classification, indicating a very high level of diagnostic potential. Additionally, to prove the speech biomarker’s reliability as a physiological indicator of PD, Hajjar et al. analyzed digital speech biomarkers using ML-based techniques [16]. The researchers found a correlation between speech features and clinical data analyzed from traditional biomarkers. They collected connected speech, neuropsychological, neuroimaging, and CSF AD biomarker data from 92 cognitively unimpaired and 114 impaired participants. The participants were classified based on amyloid beta positivity and negativity. After analyzing the lexical–semantic and acoustic features, each feature showed AUC scores of 0.80 and 0.77 in detecting MCI. Additionally, the researchers compared the results with brain connectivity computed via rs-fMRI data in Figure 5C,D. With the correlation analysis of connectivity and speech biomarkers, this study suggested that speech biomarkers may identify cognitive impairment in the early stage and predict disease progression.

While speech features could serve as digital biomarkers for ND, Rios-Urrego et al. proposed an automatic speech-based assessment for diagnosing PD using machine learning [145]. Diagnosing through speech shows promise for detecting cognitive dysfunction and motor impairments, which are common in NDs. However, a significant limitation arises in applying a universal method due to linguistic diversity across countries and regions. Rios-Urrego et al. aimed to overcome these language differences and develop automated diagnostic technology to enable early diagnosis and continuous monitoring based on speech. They trained ML models in German and Spanish and used them to differentiate between PD, essential tremors (ETs), and HCs in Czech. In distinguishing PD from ETs, they achieved up to 86% AUC, and when adding HCs for a tri-class classification, they reached up to 72% AUC. These results demonstrate that developing a universal machine learning model for speech features can address linguistic variability and provide excellent diagnostic, predictive, and monitoring performance.

### 4.6. Multimodal Analysis of Digital Biomarker

Digital biomarkers can be used together for multimodal analysis to increase diagnosis performance. Only a few studies have tried to analyze different types of digital biomarkers together. However, the results of recent studies emphasized that the convergence of modalities is effective in diagnosing ND.

**Figure 6:**
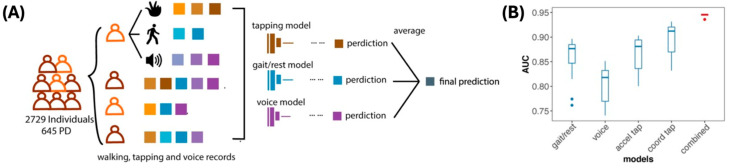
Recent results of multimodal analysis of digital biomarkers for ND diagnosis. (**A**) Individual prediction models were developed with finger-tapping, gait, and speech data, and the models were integrated [83]. (**B**) The integrated model showed a significantly higher AUC score than the individual models.

Deng et al. collected gait, finger-tapping, and speech data from 2729 individuals, including 645 PD patients [83]. They developed prediction models based on finger tapping, gait, and speech and then created a final model that integrated all the individual models (Figure 6A). The individual models could distinguish PD patients from HCs, and the AUC scores for tapping, gait, and speech models are 0.692, 0.8983, and 0.8335, respectively. After the researchers built the models, they incorporated those individual models into cross-validation and performance enhancement; the integrated model could discriminate PD patients with a significantly increased AUC score of 0.944 (Figure 6B).

Recently, Ouyang et al. built a multimodal analysis system for AD diagnosis, confirming that integrating digital biomarkers could significantly enhance diagnostic accuracy [146]. They collected biomarkers from 22 activities involving 31 AD patients, 30 MCI patients, and 30 HC patients, utilizing a depth camera, audio recording, and radar sensor. Their results showed that integrating biomarkers could increase the accuracy of AD and MCI discrimination from HCs, and the accuracy improves with more modalities in the prediction model. Although this study did not utilize any digital biomarkers categorized in this review, the findings emphasized the importance of cross-validation and integrating various biomarkers.

## 5. Digital Biomarkers with Traditional Biomarkers and ML Analysis

Although developed digital biomarkers show promise in diagnosing ND, traditional biomarkers remain the most reliable and widely used method. Researchers are exploring potential digital biomarkers that promise to be powerful, noninvasive, and highly accurate tools for diagnosing various neural diseases. However, ND diagnosis must confirm the presence of the illness and analyze and monitor factors such as the current severity of the condition, progression, and response to treatment [147]. Because digital biomarkers focus on disease symptoms, they have limitations in quantifying, monitoring, and diagnosing the onset stage of ND. To address this, the use of traditional biomarkers alongside digital biomarkers is emerging. Due to the complex feature analysis of digital biomarkers and multimodal analysis with traditional biomarkers, ML-based analysis is sufficient in this approach.

A recent study examined the diagnosis of AD and MCI using EEG feature-based analysis [81]. Jiao et al. incorporated traditional and digital biomarkers, such as APOE type, amyloid beta, and tau. The research involved a group of 1077 individuals, comprising 246 HCs, 125 patients with other forms of dementia, 330 AD patients, and 376 MCI patients. Findings indicated that CSF analysis alone could classify HCs, MCI, and AD with a 70% accuracy rate. Optimizing EEG analysis resulted in slightly improved disease onset and progression predictions compared to CSF and APOE analysis alone. However, the EEG, CSF, and APOE integrated prediction methods showed the most accurate predictions. The study concluded that combining these biomarkers can yield a more precise diagnosis of AD and MCI.

Additionally, Bayat et al. suggested GPS-based driving behavior as a digital biomarker for preclinical AD and conducted integrated prediction results of the proposed digital biomarker and APOE status [104]. They continuously recorded dates, times, latitude and longitude coordinates, and speeds using GPS for each group over a year. They then conducted ML analysis to diagnose the entire group, achieving an average accuracy of 89%. Furthermore, when age and APOE status were combined with driving features, the diagnostic accuracy increased significantly to 96%.

The convergence of digital and traditional biomarkers offers additional advantages, significantly enhancing accuracy in diagnosing ND. Brzenczek et al. recently proposed an integrated prediction method for PD based on gait analysis, supplemented by omics and patient clinical data [128]. Researchers developed a cross-validated ML model for diagnosis, severity assessment, and detection of mobility impairments while predicting comorbidities, non-motor outcomes, and progression speed. Since gait analysis relies on gait impairment, unimodal models based on gait data struggle to predict non-motor outcomes and disease progression. The integrated model demonstrated significant accuracy of AUC 0.83 to 0.92 for PD compared to HCs and up to 0.75 for severity classification. This indicates that the convergence of digital and traditional biomarkers, along with ML analysis, can improve the applications of digital biomarkers and enhance the performance of diagnosis and prediction monitoring.

Although only a few studies reported the integrated prediction method of digital and traditional biomarkers, the need for integrated studies with traditional biomarkers and ML/DL methods is still emerging due to the necessity of precise diagnosis and prediction via digital biomarkers [18,84,148,149].

## 6. Conclusions and Future Perspective

This review summarizes the advancements in traditional and digital biomarkers for ND, emphasizing their strengths and limitations. Traditional biomarkers, such as CSF biomarkers and brain imaging (fMRI, PET), offer high sensitivity and quantification capabilities, making them invaluable for clinical decision-making. However, their reliance on invasive procedures, expensive infrastructure, and limited applicability for continuous or personalized monitoring pose significant challenges in meeting the growing demand for accessible diagnostic tools.

Conversely, digital biomarkers have emerged as a promising alternative. They leverage phenotypic changes to reflect cognitive and motor dysfunctions associated with NDs. Features derived from EEG, eye movement, gait analysis, finger tapping, and speech have demonstrated substantial potential in ND diagnosis. While individual digital biomarkers show promise, integrating multiple biomarkers into a unified diagnostic framework could significantly enhance diagnostic accuracy and reliability.

Despite these advancements, the current reliance on validation using patients with pre-established clinical diagnoses or traditional biomarker analyses underscores a critical gap. For digital biomarkers to gain widespread clinical adoption, rigorous validation through blind testing and prospective cohort studies is imperative. Such efforts would establish standalone efficacy and enable broader applications beyond traditional settings.

Looking forward, the synergy between traditional and digital biomarkers offers a transformative approach to ND diagnosis and management. Validated digital biomarkers, used with traditional biomarkers, can enable early prediction, improve diagnostic precision, and facilitate real-time disease progression and therapeutic efficacy monitoring. Furthermore, integrating digital biomarkers into point-of-care systems and wearable technologies can democratize access to ND diagnostics, making personalized monitoring feasible across diverse populations and settings.

As the field advances, interdisciplinary collaboration between clinicians, engineers, and data scientists will be crucial to overcoming current limitations and unlocking the full potential of digital biomarkers. By addressing challenges in validation, integration, and accessibility, the future of ND diagnosis and management promises to be more proactive, precise, and patient-centered.

## Figures and Tables

**Table 1:** Traditional biomarkers of AD and PD.

Biomarker	Disease	Type	CSF Level *	Application			References
				Early Diagnosis	Prognosis	Monitoring	
Apolipoprotein E (APOE)	AD, PD	Genotype	-	O			[29,36,37]
T-Tau	AD, PD	CSF	↑	O	O	O	[38,39,40,41]
P-Tau			↑	O	O	O	[38,39,40,41,42,43]
BACE1			↑	O			[44,45]
α-Synuclein			↑ (AD) ↓ (PD)		O		[41,46,47,48]
UCH-L1	AD, PD		↑ (AD) ↓ (PD)			O	[49,50,51]
YKL-40	AD, PD		↑ (AD) ↓ (PD)	O	O	O	[52,53,54]
Rs-fMRI	AD, PD	Brain image	-	O		O	[55,56]
FDG-PET	AD		-	O			[57,58,59]
Aβ42	AD, PD	CSF • Brain image	↓	O	O	O	[29,38,42,43,46,60,61,62]
Aβ42/Aβ40 ratio			↓	O	O	O	
Neurofilament light chain (NfL)			↑		O		[63,64,65,66,67,68]
Dopamine/DOPAC	PD		↓	O		O	[69,70,71,72,73,74]

* Changes in CSF biomarker level in patients: ↑, increase; ↓, decrease.

**Table 2:** Digital biomarkers for the diagnosis of ND.

Method	Potential Biomarkers	Measurement		Real-Time Monitoring *	References
		Device	Limitation		
EEG	Relative band power, signal complexity, functional connectivity	EEG electrode	Sufficient device wearability	O	[87,88,89,90,91,92,93,94,95,96,97,98,99,100]
Sleep behavior	Total sleep time, sleep efficiency, REM sleep, REM latency	EEG electrode, EMG electrode, accelerometer, smartwatch	Sleep-dependent measurement	Δ	[101,102,103]
Driving behavior	Driving space, driving performance	GPS	Driving-dependent measurement	Δ	[104]
Eye movement	Saccade, blink, fixation, pupil size, latency, gain, correctness	Camera	Task-dependent measurement	Δ	[105,106,107,108,109,110,111,112,113,114,115,116,117]
Gait	Step duration, stance, swing, velocity, variability, symmetry, amplitude, double support	Camera, accelerometer, gyroscope	Sufficient device wearability or location-specific measurement	O	[17,118,119,120,121,122,123,124,125,126,127,128]
Finger tapping	Tapping frequency, inter-tap interval, variability	Smartphone, camera, wearable electrode	Task-dependent measurement	Δ	[129,130,131,132,133,134,135,136,137]
Speech	Acoustic features, lexical–sematic features	Mic, smartphone	Language variability	O	[16,138,139,140,141,142,143,144,145]

Abbreviations: REM, rapid eye movement; GPS, global positioning system. * Applicability: O, applicable; Δ, promising.

## Data Availability

The data presented in this study are available from the corresponding authors upon request.
